# DMD-10 is dispensable for the initial development of amphid sensory neurons and their survival in mature *C. elegans*

**DOI:** 10.17912/micropub.biology.000408

**Published:** 2021-06-14

**Authors:** Irene Nguyen, Eric S. Luth

**Affiliations:** 1 Simmons University, Department of Biology, Boston, MA

## Abstract

Mechanosensory or chemosensory activation of glutamatergic**ASH amphid sensory neurons promotes avoidance**behaviors in *C. elegans*. Worms**with mutations in the transcription factor DMD-10 have impaired ASH-mediated sensorimotor reflexes. We hypothesized that the behavioral dysfunction in *dmd-10 *mutants could arise from impaired ASH development or survival leading to disrupted glutamatergic signaling.**To test this, we performed *in vivo* fluorescence microscopy of young adult *C. elegans *amphid neurons after labeling with the lipophilic dye DiI. We quantified the number of ASH neurons as well as five other amphid sensory neuron pairs. We found that the number of amphid neurons in *dmd-10 *mutants was the same as in wild-type worms. Our results suggest that *dmd-10 *is not required for amphid neuron development or survival in mature *C. elegans.*

**Figure 1. dmd-10 mutant worms display the typical number of amphid sensory neurons pairs:**
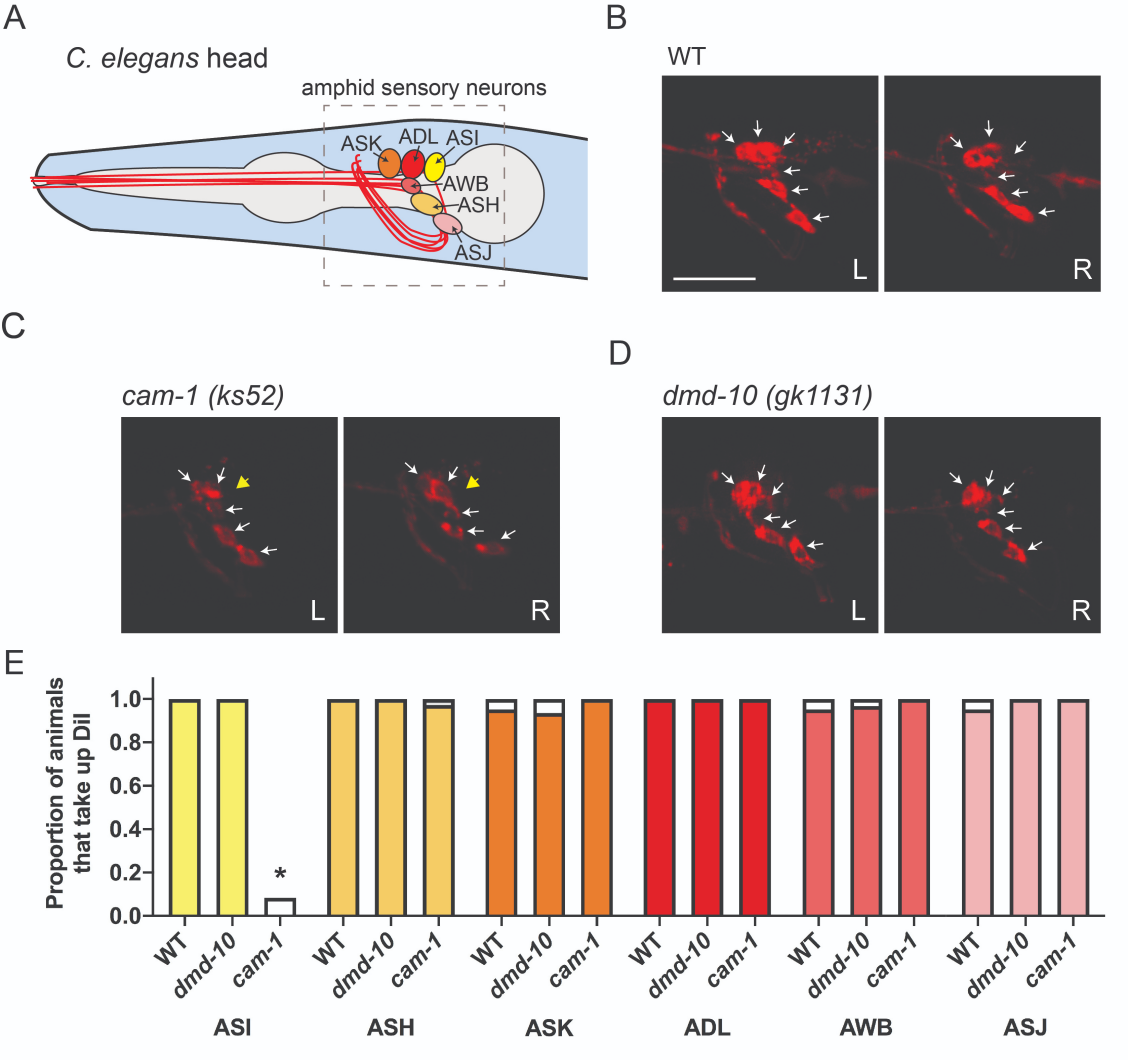
(A) Map of the positions of *C. elegans* ASH, ADL, ASI, AWB, ASK, and ASJ sensory neurons. (B-D) DiI labeled ASH, ASI, ADL, ASK, AWB, and ASJ sensory neurons of a representative wildtype (WT)(B) *cam-1* (C) and *dmd-10* (D) wormvisualized via fluorescence microscopy. Images show the presence of each fluorescently-labeled sensory neuron cell body (white solid arrows) in both the left (L) and right (R) sets. Yellow arrowheads note missing ASI cell bodies in *cam-1* worms. (E) Graphs show the proportion worms with DiI filled amphid neurons for each genotype (WT n=34, *cam-1* n=36, *dmd-10* n=31). Unfilled portion of each bar represents proportion of worms with 1 out of 2 neurons observed. *=p<0.05 Chi-squared test, *cam-1* compared to wildtype.

## Description

The *C elegans* transcription factor DMD-10 is an ortholog of the human DBRTB, a Doublesex/MAB-3(DM)-related transcription factor. Young adult *dmd-10* *(gk1131)* loss-of-function mutant *C.* *elegans* exhibit selective defects in sensorimotor behaviors (Durbeck *et al.* 2021). They are less responsive to nose-touch mechanosensory stimulation and high osmolarity, both of which are transduced by a pair of ASH sensory neurons (Kaplan and Horvitz 1993; Bargmann 2006; Lindsay *et al.* 2011) *dmd-10* mutants also show an approximately 50% decrease in their behavioral response to direct, optogenetic activation of channelrhodopsin-2 expressed under the control of promoters whose expression patterns overlap only in ASH (Ezcurra *et al.* 2011; Durbeck *et al.* 2021). Considering the known effects of other DM domain-containing proteins on neuronal development (Tresser *et al.* 2010; Yoshizawa *et al.* 2011; Saulnier *et al.* 2013), one potential explanation for this could be that one of the two ASH neurons does not develop properly or survive in adult *dmd-10* mutant animals.

We set out to determine whether the number of ASH neurons is altered in *dmd-10* mutants. The left and right pairs of ASH sensory neurons and five other nearby ciliated sensory neuron pairs (ASI, ADL, ASK, AWB, and ASJ) are found in the amphids, a pair of sensilla in the heads of nematodes like *C. elegans* (Inglis *et al.* 2007). These cells can all be labeled with the lipophilic fluorescent dye DiI (1,1-Dioctadecyl-3,3,3’,3’-tetramethylindocarbocynanine perchlorate) and identified based on their stereotyped position (Shaham 2006) (Fig. 1A). DiI intercalates into the lipids of the ciliated dendrites of sensory neurons that protrude outside the worm cuticle and ultimately labels the entire plasma membrane of these cells (Collet *et al.* 1998; Schultz and Gumienny 2012). Because recent tissue-specific and single-cell gene expression studies indicate that *dmd-10* may be expressed in ciliated sensory neurons including ASK (Cao *et al.* 2018; Packer *et al.* 2019), we expanded our analysis to quantify all amphid sensory neurons that are labeled with DiI.

To test our hypothesis that DMD-10 is required for formation or survival of amphid sensory neurons, we incubated N2 wildtype and *dmd-10* mutant young adult worms with DiI and performed *in vivo* fluorescence microscopy. We chose to examine young adult worms because behavioral defects were previously observed in this developmental stage (Durbeck *et al.*. 2021). To determine whether our methods were reliable enough to detect the absence of a single amphid neuron or a pair of amphid neurons in DiI-labeled worms, we similarly imaged *cam-1/kin-8* *(ks52)* mutant worms. Consistent with a previous report (Koga *et al.* 1999), we saw that *cam-1/kin-8* mutants had a specific dye-filling defect in ASI neurons (Fig. 1C, E).

Fluorescence imaging of *dmd-10 (gk1131)* mutants revealed no differences in the number of ASH sensory neurons between the N2 wildtype (WT) and *dmd-10* mutant worms (Fig. 1B, D, E). We also found no differences in the number of ADL, ASI, AWB, ASK, or ASJ amphid sensory neurons (Fig. 1B, D, E). We observed all six of these sensory neurons in both the left and right side of the head of *dmd-10* mutants (Figure 1D, E). This indicates that there were no gross defects in the presence or morphology of amphid neurons and suggests that mutation of *dmd-10* does not affect amphid neuron development or survival into young adulthood.

*Dmd-10* mutants have defects in ASH-dependent behaviors (Durbek *et al.* 2021), but our data suggests this cannot be attributed to impaired ASH development or survival. We found that amphid sensory neurons of *dmd-10* mutants appear in the stereotypical, wildtype positions and appropriately extend ciliated dendrites into the external environment. It remains possible that, despite their unaltered gross morphology, ASH neurons in *dmd-10* mutants may be functionally impaired. Future studies will be needed to identify the function of *dmd-10* in ASH and in other amphid neurons in which it is expressed (Cao *et al.* 2018; Packer *et al.* 2019). Because other *C. elegans* DMD family members have been shown to regulate sexually-dimorphic neuronal connectivity (Oren-Suissa *et al.* 2016, Serrano-Saiz *et al.* 2017), it will also be interesting to examine differences in sensory neuron function and morphology between hermaphrodites and males.

## Methods

*C. elegans* strains were housed at 20˚C and maintained according to standard procedures (Brenner 1974). N2, *dmd-10 (gk1131),* and *cam-1 (ks52)* loss of function mutant worms were used. Young adult worms, defined as adults lacking visible eggs in the uterus, were incubated for 3 h in 1 ml of M9 buffer (KH_2_PO_4_, 22.0 mM; Na_2_HPO_4_, 42.3 mM; NaCl, 85.6 mM autoclaved, plus 1 mM sterile MgSO_4_) to which 5 μl of 2 mg/ml DiI (1,1-Dioctadecyl-3,3,3’,3’-tetramethylindocarbocynanine perchlorate, Sigma #468495) was added (Shaham 2006). After washing worm suspensions with M9 twice, worms were mounted on 2% agarose pads containing 3.4 mM levamisole (Sigma #L9756). Laterally-oriented worms were imaged at 400x on an Axio Observer microscope equipped with an Apotome 2 optical sectioning device (Carl Zeiss) and illuminated with a Colibri 7 LED at 567 nm at 70% intensity for 50-60 ms. A Cy3 emission filter was used. Amphid neurons were counted after collecting 30-35 µm Z-stacks with a 0.4 µM interval using an Orca-4 camera (Hamamatsu). Orthogonal projections of left and right amphid neurons were created for display.

## Reagents

**Table:** 

**Strain**	**Genotype**	**Available from**
N2	*Caenorhabditis elegans*	CGC
VC2341	*dmd-10 (gk1131) V*	CGC
FK163	*cam-1 (ks52) II*	CGC
